# Gut microbiome and psychiatric disorders

**DOI:** 10.1186/s12888-023-05003-4

**Published:** 2023-07-07

**Authors:** Kazi Farhana Afroz, Mirko Manchia

**Affiliations:** 1 Department of Pharmacology, Emory University, Atlanta, GA USA; 2 Section of Psychiatry, Department of Medical Sciences and Public Health, University of Cagliari, Cagliari, 09121 Italy; 3 Unit of Clinical Psychiatry, University Hospital Agency of Cagliari, Cagliari, 09121 Italy; 4 Department of Pharmacology, Dalhousie University, Halifax, NS B3H 0A2 Canada

## Abstract

Several pieces of evidence show that gut microbiota can impact psychiatric disorders. However, no mechanism behind the relationship has been identified. Host genetics and their diets have a significant impact on the gut microbiota. More advanced studies are needed to find the mechanism and develop new therapeutic strategies.

In the last thirty years, there have been considerable advancements in the comprehension of the interplay between the biological environment of the gut and the central nervous system (CNS). This biological and functional connection has been first observed and described in scientific terms at the end of the 19th century, with seminal reports from a series of authors (summarized in Aziz and Thomson [1]) on the changes in intestinal motility determined by modification of the emotional status. These physiological changes are modulated by the activity of the nervous system which in turn is regulated by variation in neurochemical circuitry. Of note, the synthesis of key neurotransmitters is substantially influenced by the microbial flora of the gut, with several bacterial strains producing monoamines [2]. It is not surprising that the evidence cumulated on the link between gut microbiota and emotional and behavioral regulation has led to the analysis of its involvement in modulating the liability to severe psychiatric disorders [3]. For instance, a recent review highlighted that across the three most severe psychiatric disorders (schizophrenia, bipolar disorder, and major depressive disorder) there are consistent alterations of the gut microbiota with higher relative abundances of the genera *Streptococcus*, *Lactobacillus*, and *Eggerthella* and lower relative abundance of the butyrate-producing *Faecalibacterium* [4]. Interestingly, all these three increased genera were associated with higher symptom severity [4]. These observed associations, which still do not imply causality, appear evident even in prodromal stages of severe psychiatric disorders [5] or in more severe clinical forms such as those characterized by treatment resistance (TR) [6–8]. The latter studies pointed to several gut biomarker candidates such as *Clostridium perfringens* species for TR in anxiety and depression [6], *Actynomyces* and *Porphyromonas* genera for TR in schizophrenia [7], and *Proteobacteria, Tenericutes* phyla, and family *Peptostreptococcaceae* for TR in major depressive disorder [8].

Despite the increasing amount of evidence, there are still several gaps in the comprehension of the role of the gut microbiota in predisposing toward the development of psychopathology and on the magnitude of this effect. One key unresolved question is whether alterations of gut microbiota can be predictive of subsequent clinical manifestations of the disorders. This is related to one of the properties of gut microbiota, consisting in its adaptability to environmental stimuli and changes, that is based on its dynamic genomic constitution. This, however, renders the metagenomic of gut microbiota unstable over time, and even more when specific environmental factors, such as pharmacological treatments, are present. Even so, there is interesting data showing that studies with accurate longitudinal designs could shed light on the causal relationship between gut microbiota variation and psychopathological manifestations. In a recent study of a longitudinal pre-birth cohort of 213 mothers and 215 children, alpha diversity of the maternal faecal microbiota during the third trimester of pregnancy predicted child internalising behaviour at two years of age [9]. Interestingly, taxa from butyrate-producing families, *Lachnospiraceae* and *Ruminococcaceae*, were more abundant in mothers of children with normative behavior [9].

Since the early 20th century, several pieces of evidence have shown that the formulation of more than one probiotic bacterial strain can improve mental health conditions. Administration of four Bifidobacterium strains in germ-free mice elucidated improvement in synaptic density, neuronal activity, and reduced microglial activity. However, any specific mechanisms by which the gut microbial population is involved in the pathophysiology of psychiatric disorders are still unknown. A recent systematic review and meta-analysis of gut microbiome studies in adults from several major psychiatric disorders reported a decrease in the anti-inflammatory short-chain fatty acid (SCFA) producing bacterial genera while an increase in the pro-inflammatory genera in individuals with psychosis and schizophrenia, major depressive disorder, bipolar disorder, and anxiety [10]. In all these individuals, the relative abundance of Faecalibacterium and Coprococcus decreased whereas Eggerthella abundance was increased. Moreover, another study reported that Anaeromassilibacillus, Gordonibacter, and Parasutterella genera were most significantly different in people with a social anxiety disorder (SAD) compared to healthy individuals [11]. In addition, Afroz et al. 2021, reported an association between maternal high salt diet-induced gut microbial alteration with autism spectrum disorder (ASD)-like behavior in the offspring [12]. The paper suggested that if the maternal gut microbiome is altered due to their dietary habits, it can impact the neurodevelopment of the offspring.

A major way of involvement of gut microbiome in psychiatric disorders is by regulating the host genetics. For several years a two-way interaction between the gut microbiota and host genetics was reported. The gut microbiota can impact the transcription factor binding which ultimately impacts the chromatin accessibility in the host’s body [13]. Studies reported that alteration of microglial activation genes and p28 MAPK signaling pathway genes were associated with social behavior in rodent model animals [14]. Alongside, the gut microbiota alters histone acetylation and methylation which results in host epigenetic changes [15].

All the previous studies described earlier confirm the impacts of gut microbial alteration on the psychiatric disorder pathology in the host organisms. More in-depth studies are needed to identify the exact mechanism by which the gut microbiota affects the host’s mental health. Knowledge about the role of gut microbiota is important to develop new therapeutic strategies for psychiatric disorders. As the type of diet modulates the gut microbial population, changing dietary habits as well as consumption of probiotic supplements could be beneficial for psychiatric disease alleviation. Currently, most data about the effects of gut microbiota on psychiatric diseases are based on animal studies. Therefore, more clinical studies are required to find effective therapeutic strategies.

## Data Availability

Not applicable.

## Abbreviations

CNS: Central nervous system
TR: Treatment resistance
SCFA: Short-chain fatty acid
SAD: Social anxiety disorder
ASD: Autism spectrum disorder
